# The prognosis analysis of different metastasis pattern in patients with different breast cancer subtypes: a SEER based study

**DOI:** 10.18632/oncotarget.14300

**Published:** 2016-12-27

**Authors:** Haiyong Wang, Chenyue Zhang, Jingze Zhang, Li Kong, Hui Zhu, Jinming Yu

**Affiliations:** ^1^ Department of Radiation Oncology, Shandong Cancer Hospital Affiliated to Shandong University, Shandong Academy of Medical Sciences, Shandong 250117, China; ^2^ Department of Integrative Oncology, Fudan University Shanghai Cancer Center, Shanghai 200032, China

**Keywords:** breast cancer, metastasis pattern, prognosis, breast cancer subtypes, SEER

## Abstract

Studies on prognosis of different metastasis patterns in patients with different breast cancer subtypes (BCS) are limited. Therefore, we identified 7862 breast cancer patients with distant metastasis from 2010 to 2013 using Surveillance, Epidemiology, wand End Results (SEER) population-based data. The results showed that bone was the most common metastatic site and brain was the least common metastatic site, and the patients with HR+/HER2− occupied the highest metastasis proportion, the lowest metastasis proportion were found in HR-/HER2+ patients. Univariate and multivariate logistic regression analysis were used to analyze the association, and it was found that there were significant differences of distant metastasis patterns in patients with different BCS(different *P* value). Importantly, univariate and multivariate Cox regression analysis were used to analyze the prognosis. It was proven that only bone metastasis was not a prognostic factor in the HR+/HER2-, HR+/HER2+ and HR-/HER2+ subgroup (all, *P* > 0.05), and patients with brain metastasis had the worst cancer specific survival (CSS) in all the subgroups of BCS (all, *P*<0.01). Interestingly, for patients with two metastatic sites, those with bone and lung metastasis had best CSS in the HR+/HER2- (*P*<0.001) and HR+/HER2+ subgroups (*P*=0.009) However, for patients with three and four metastatic sites, there was no statistical difference in their CSS (all, *P*>0.05).

## INTRODUCTION

Breast cancer is the most frequently diagnosed cancer and the leading cause of cancer death among females worldwide, with an estimated 1.7 million incidence and 521,900 mortalities in 2012 [1]. Importantly, breast cancer alone accounts for 25% of all cancers and 15% of all cancer-related deaths among females [1]. About 90% of the deaths can be attributed to the metastasis during treatment [2, 3]. It has been reported that about 20%-30% of breast patients developed distant metastasis upon initial diagnosis and treatment [4, 5]. Bone, liver, lung and brain are the proper niches suitable for metastasis in breast cancer patients [6, 7].

Factors such as tumor size, nodal involvement, histologic grade and hormone receptor status can effectively influence the occurrence and progression of breast cancer metastasis [8, 9]. However, the relationship between clinical related factors and the exact patterns of distant metastasis is not well established. According to the expression status of progesterone receptor (PR), estrogen receptor (ER) and human epidermal growth factor receptor 2 (HER2), breast cancer can be divided into four major subtypes: hormone receptor (HR)+/HER2−, HR+/HER2+, HR−/HER2+ and HR−/HER2−, as previous studies have reported [10, 11]. Breast cancer patients with different subtypes have demonstrated discrepancies in responses to a wealth of therapies including radiotherapy, chemotherapy and targeted therapy, which may have an influence on disease recurrence [12, 13]. However, studies exploring the relationship between BCS and the distant metastasis patterns are limited and inconsistent by far. Importantly, does prognosis differ for the patients with the same metastatic site according to different BCS? Also, does prognosis differ for the patients with the same BCS according to different metastatic sites? However, no relevant studies have been reported regarding these issues.

In the present study, using the Surveillance, Epidemiology, and End Results (SEER)-registered database, we analyzed the relationships between the BCS and the exact distant metastasis patterns. Importantly, we mainly analyzed the prognosis in patients with the same metastatic pattern according to different BCS, and the patients with the same BCS according to different metastatic patterns.

## RESULTS

### Patient demographics

There were 7862 female breast cancer patients reported in the SEER database from 2010 to 2013. The clinical characteristics and pathological features of all the patients were summarized in Table 1. Most patients were diagnosed at the age of more than 50-year-old (77.2%). Most patients were white race (75.0%). 33.3% of patients were diagnosed at Grade II and 42% of patients were diagnosed at Grade III. In addition, the proportion of patients with ER positive, PR positive and HER2 negative was 74%, 59.4% and 74.1% respectively. Interestingly, 59.2% of patients were HR+/HER2- based on the BCS. In addition, bone was the most common metastatic site (66.3%), and brain was the least common metastatic site (7.3%). The detailed characteristics were shown in Table 1.

**Table 1:** Characteristics of breast cancer patients with AJCC stage IV from SEER Database from 2010-2013

Characteristics	Number	%
**Age**		
< 35	270	3.4
35-49	1521	19.3
≥ 50	6071	77.2
**Race**		
White	5900	75.0
Blank	1336	17.0
Others	626	8.0
**Grade**		
I	468	6.0
II	2620	33.3
III	3302	42.0
Unknown	1472	18.6
**ER status**		
Positive	5825	74
Negative	2037	25.9
**PR status**		
Positive	4673	59.4
Negative	3189	40.6
**HER2 status**		
Positive	2036	25.9
Negative	5826	74.1
**Breast cancer subtype**		
HR+/HER2-	4652	59.2
HR+/HER2+	1310	16.7
HR-/HER2+	726	9.2
HR-/HER2-	1174	14.9
**Metastatic site**		
Bone	5213	66.3
Brain	574	7.3
Liver	2053	26.1
Lung	2380	30.3
Others	931	11.8

### Metastasis pattern based on different BCS

**Figure 1:**
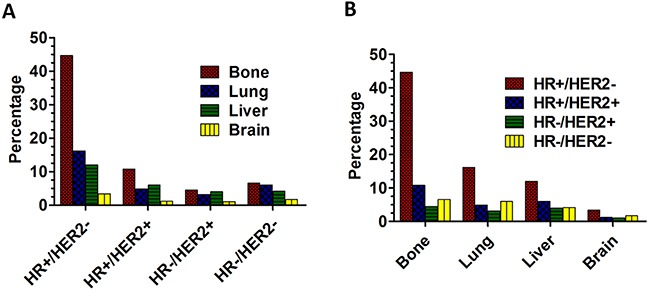
The percentage of distant metastasis sites **A**. The percentage of distant metastasis sites based on different BCS. **B**. The percentage of BCS based on different distant metastasis sites.

The distant metastatic sites were concluded as follows: bone metastasis, lung metastasis, liver metastasis and brain metastasis. In addition, the BCS were classified into the following subgroups: HR+/HER2−, HR+/HER2+, HR−/HER2+ and HR−/HER2-. Figure 1A showed that bone was the most common metastatic site and brain was the least common metastatic site in all the BCS. In patients with HR+/HER2−, the percentage of patients with metastasis to bone, lung, liver and brain was 44.7%, 16.2%, 12.0% and 3.4% ; In the HR+/HER2+ patients, the percentage was 10.8%, 4.9%, 6.0%, 1.2%; In the HR-/HER2+ patients, the percentage was 4.5%, 3.2%, 4.0%, 1.0%, while in those patients with HR-/HER2−, the percentage was 6.6%, 6.0%, 4.2%, 1.7%. Figure 1B showed that the patients with HR+/HER2− were most likely to develop metastasis, and the patients with HR-/HER2+ were less likely to develop metastasis regardless of metastatic pattern. In patients with bone metastasis, a bulk of them were HR+/HER2−, accounting for 44.7%, and those with HR-/HER2+ accounts for a mere 4.5%; In patients with lung metastasis, the percentage were HR+/HER2− (16.2%), HR+/HER2+ (4.9%), HR-/HER2+ (3.2%), HR-/HER2− (6.0%); In patients with liver metastasis, the percentage were HR+/HER2− (12.0%), HR+/HER2+ (6.0%), HR-/HER2+ (4.0%), HR-/HER2− (4.2%). The percentage of patients with brain metastasis were HR+/HER2− (3.4%), HR+/HER2+ (1.2%), HR-/HER2+ (1.0%), HR-/HER2− (1.7%).

### Combination metastasis analysis based on different BCS

**Figure 2:**
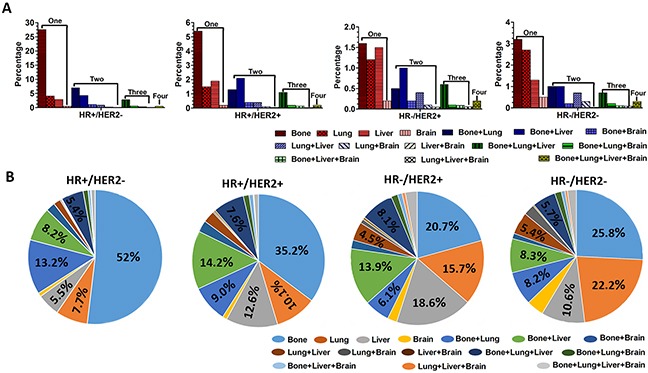
The percentage of distant combination metastasis **A**. The percentage of different distant combination metastasis based on different BCS in all the patients. **B**. The percentage of all different distant combination metastasis in patients with specific BCS.

Interestingly, many breast cancer patients developed more than one metastatic site. All the possible combinations of metastasis patterns were summarized in Table 2. The results showed that about 27.6% of patients with HR+/HER2−, 5.2% of patients with HR+/HER2+, 1.6% of patients with HR-/HER2+, 3.2% of patients with HR-/HER2− only had bone metastasis. Mere liver metastasis or brain metastasis were seldom seen in all BCS. The most common two-site combination metastasis was different among four BCS. In patients with HR+/HER2−, the most common metastasis of two-site combination metastasis was bone and lung (7.0%); In patients with HR+/HER2+, it was bone and liver (2.1%); In patients with HR-/HER2+, it was bone and liver (1.1%); In patients with HR-/HER2−, it was bone and lung or bone and liver (1.0% for both). The most common three-site combination metastasis was bone, lung and liver in all the four BCS. The percentage was 2.8% (HR+/HER2-), 1.1% (HR+/HER2+), 0.6% (HR-/HER2+), and 0.7% (HR-/HER2-) respectively. Metastasis to four sites was rare, the percentage of which was below 1% in all the four BCS. The detailed statistical results were shown in Figure 2A and Table 2.

**Table 2:** Frequencies of combination metastasis sites in breast cancer patients with AJCC stage IV based on different BCS

Metastasis site	HR+/HER2-	HR+/HER2+	HR-/HER2+	HR-/HER2-	P value
	Number (%)	Number (%)	Number (%)	Number (%)	
**Only one site**					*P* < 0.001
Bone	2172 (27.6)	412 (5.2)	128 (1.6)	248 (3.2)	
Lung	323 (4.1)	118 (1.5)	97 (1.2)	214 (2.7)	
Liver	228 (2.9)	148 (1.9)	115 (1.5)	102 (1.3)	
Brain	37 (0.5)	13 (0.2)	14 (0.2)	40 (0.5)	
**Two sites**					*P* < 0.001
Bone+Lung	553 (7.0)	106 (1.3)	38 (0.5)	79 (1.0)	
Bone+Liver	341 (4.3)	167 (2.1)	86 (1.1)	80 (1.0)	
Bone+Brain	90 (1.1)	33 (0.4)	13 (0.2)	13 (0.2)	
Lung+Liver	71 (0.9)	34 (0.4)	28 (0.4)	52 (0.7)	
Lung+Brain	12 (0.2)	4 (0.05)	6 (0.08)	26 (0.3)	
Liver+Brain	8 (0.1)	5 (0.06)	4 (0.05)	3 (0.04)	
**Three sites**					*P* = 0.098
Bone+Lung+Liver	224 (2.8)	89 (1.1)	50 (0.6)	55 (0.7)	
Bone+Lung+Brain	49 (0.6)	16 (0.2)	10 (0.1)	15 (0.2)	
Bone+Liver+Brain	24 (0.3)	12 (0.2)	7 (0.09)	9 (0.1)	
Liver+Lung+Brain	4 (0.05)	2 (0.03)	5 (0.06)	6 (0.08)	
**Four sites**					*P* < 0.001
Bone+Lung+Liver+Brain	41 (0.5)	13 (0.2)	18 (0.2)	21 (0.3)	

Next, we analyzed the different combination metastasis in patients with specific BCS. As we expected, the results showed the most common combination metastasis was “single bone metastasis”, with the percentage of 52% in patients with HR+/HER2-, 35.2% in patients with HR+/HER2+, 20.7% in patients with HR-/HER2+, 25.8% in patients with HR-/HER2-, respectively. Interestingly, the second common combination metastasis was different among four BCS. In patients with HR+/HER2-, the combination metastasis was bone and lung (13.2%); In patients with HR+/HER2+, the combination metastasis was bone and liver (14.2%); In patients with HR-/HER2+, the combination metastasis was only liver (18.6%); In patients with HR-/HER2-, the combination metastasis was only lung (22..2%). The detailed statistical results were shown in Figure 2B.

### The association between the sites of distant metastasis and BCS

To further evaluate the relationship between the metastasis pattern and BCS, univariate and multivariate logistic regression analysis were used to analyze the association.

For bone metastasis, the results showed that the patients with HR+/HER2− had a significantly higher probability than those with the other three BCS (HR+/HER2+ vs HR+/HER2-: OR 0.699, CI% 0.584-0.765; HR-/HER2+ vs HR+/HER2-: 0.355, CI% 0.301-0.419; HR-/HER2- vs HR+/HER2-: 0.323, CI% 0.281-0.372) (Table 3). For lung metastasis, the results showed that the patients with HR-/HER2− and HR-/HER2+ had a significantly higher probability than the patients with HR+/HER2− (HR-/HER2+ vs HR+/HER2-: 1.236, CI% 1.041-1.467; HR-/HER2- vs HR+/HER2-: 1.537, CI% 1.333-1.772). In addition, no significant statistical differences were found in the probability of lung metastases between the patients with HR+/HER2- and HR+/HER2+ (HR+/HER2+ vs HR+/HER2-: OR 1.013, CI% 0.881-1.164)(Table 3). For liver metastasis, the results showed that the patients with HR+/HER2- had a significantly lower probability than the patients with the other three BCS (HR+/HER2+ vs HR+/HER2-: OR 2.113, CI% 1.843-2.422; HR-/HER2+ vs HR+/HER2-: 2.864, CI% 2.420-3.390; HR-/HER2- vs HR+/HER2-: 1.413, CI% 1.211-1.648) (Table 3). For brain metastasis, the results showed that the patients with HR+/HER2− also had a significantly lower probability than the patients with the other three BCS (HR+/HER2+ vs HR+/HER2-: OR 1.371, CI% 1.073-1.751; HR-/HER2+ vs HR+/HER2-: 1.992, CI% 1.513-2.624; HR-/HER2- vs HR+/HER2-: 2.162, CI% 1.711-2.731) (Table 3).

**Table 3:** Univariate and multivariate logistic regression analysis were used to evaluate the relationship between the distant metastatic pattern and BCS

Metastasis site/Subtype	Univariate analysis		Multivariate analysis	
	Wald χ^2^	P	OR (95%CI)	P
**Bone**	498.445	< 0.001		
HR+/HER2+ vs HR+/HER2-			0.669 (0.584-0.765)	< 0.001
HR-/HER2+ vs HR+/HER2-			0.355 (0.301-0.419)	< 0.001
HR-/HER2- vs HR+/HER2-			0.323 (0.281-0.372)	< 0.001
HR-/HER2+ vs HR+/HER2+			0.531 (0.515-0.548)	< 0.001
HR-/HER2- vs HR+/HER2+			0.483 (0.481-0.486)	< 0.001
HR-/HER2- vs HR-/HER2+			0.910 (0.887-0.934)	0.653
**Lung**	75.380	< 0.001		
HR+/HER2+ vs HR+/HER2-			1.013 (0.881-1.164)	0.860
HR-/HER2+ vs HR+/HER2-			1.236 (1.041-1.467)	0.016
HR-/HER2- vs HR+/HER2-			1.537 (1.333-1.772)	< 0.001
HR-/HER2+ vs HR+/HER2+			1.220 (1.182-1.260)	0.028
HR-/HER2- vs HR+/HER2+			1.517 (1.513-1.522)	< 0.001
HR-/HER2- vs HR-/HER2+			1.243 (1.208-1.280)	0.012
**Liver**	250.503	< 0.001		
HR+/HER2+ vs HR+/HER2-			2.113 (1.843-2.422)	< 0.001
HR-/HER2+ vs HR+/HER2-			2.864 (2.420-3.390)	< 0.001
HR-/HER2- vs HR+/HER2-			1.413 (1.211-1.648)	< 0.001
HR-/HER2+ vs HR+/HER2+			1.355 (1.313-1.400)	< 0.001
HR-/HER2- vs HR+/HER2+			0.669 (0.657-0.680)	< 0.001
HR-/HER2- vs HR-/HER2+			0.494 (0.486-0.500)	< 0.001
**Brain**	57.709	< 0.001		
HR+/HER2+ vs HR+/HER2-			1.371 (1.073-1.751)	0.012
HR-/HER2+ vs HR+/HER2-			1.992 (1.513-2.624)	< 0.001
HR-/HER2- vs HR+/HER2-			2.162 (1.711-2.731)	< 0.001
HR-/HER2+ vs HR+/HER2+			1.453 (1.410-1.499)	0.005
HR-/HER2- vs HR+/HER2+			1.577 (1.560-1.595)	< 0.001
HR-/HER2- vs HR-/HER2+			1.085 (1.064-1.106)	0.505

### Survival analysis based on different metastatic pattern

Next, we regarded CSS as our primary endpoint to analyze the prognosis. Univariate analysis showed that lung, liver and brain metastasis were prognostic factors affecting CSS in all patients with four BCS (all, *P* < 0.001) (Supplementary Figure S1 and Table 4). However, bone metastasis was not a prognostic factor affecting CSS in patients with HR+/HER2- (χ2=0.085, *P*=0.770) or HR+/HER2+ (χ2=1.82, *P*=0.177) (Supplementary Figure S1 and Table 4). Further multivariate analysis showed that lung, metastasis and brain metastasis also were independent prognostic factors of CSS (all, *P* < 0.01) (Table 4). Interestingly, bone metastasis was also not an independent prognostic factor affecting CSS in patients with HR-/HER2+ (HR 0.834, CI% 0.646-1.077, *P*=0.165) (Table 4).

**Table 4:** Univariate and multivariate Cox regression analysis were used to evaluate the influence of distant metastasis sites on CSS based on different BCS

Subtype/Metastasis site	Univariate analysis		Multivariate analysis	
	Log Rank χ2	*P*	HR (95%CI)	*P*
**HR+/HER2-**				
Bone metastasis (No vs Yes)	0.085	0.770	NI	
Lung metastasis (No vs Yes)	111.75	< 0.001	0.476 (0.400-0.567)	< 0.001
Liver metastasis (No vs Yes)	158.71	< 0.001	0.526 (0.469-0.589)	< 0.001
Brain metastasis (No vs Yes)	45.17	< 0.001	0.852 (0.764-0.952)	0.005
**HR+/HER2+**				
Bone metastasis (No vs Yes)	1.82	0.177	NI	
Lung metastasis (No vs Yes)	20.163	< 0.001	0.524 (0.382-0.717)	< 0.001
Liver metastasis (No vs Yes)	52.81	< 0.001	0.449 (0.365-0.553)	< 0.001
Brain metastasis (No vs Yes)	15.75	< 0.001	0.695 (0.560-0.863)	0.001
**HR-/HER2+**				
Bone metastasis (No vs Yes)	4.11	0.043	0.834 (0.646-1.077)	0.165
Lung metastasis (No vs Yes)	28.64	< 0.001	0.461 (0.327-0.651)	< 0.001
Liver metastasis (No vs Yes)	15.58	< 0.001	0.610 (0.473-0.786)	< 0.001
Brain metastasis (No vs Yes)	20.34	< 0.001	0.612 (0.472-0.794)	0.001
**HR-/HER2-**				
Bone metastasis (No vs Yes)	15.10	< 0.001	0.737 (0.633-0.858)	< 0.001
Lung metastasis (No vs Yes)	39.94	< 0.001	0.577 (0.464-0.717)	< 0.001
Liver metastasis (No vs Yes)	29.93	< 0.001	0.639 (0.543-0.752)	< 0.001
Brain metastasis (No vs Yes)	18.54	< 0.001	0.741 (0.635-0.864)	< 0.001

NI: not included in the multivariate survival analysis.

**Figure 3:**
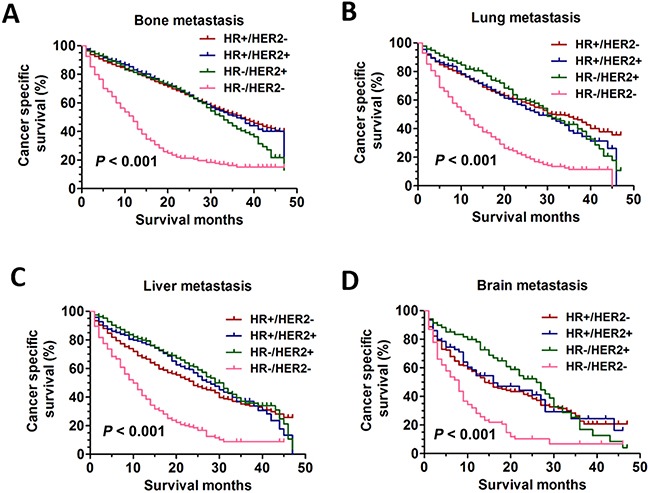
The CSS curves in breast cancer patients with different distant metastasis sites **A**. the CSS curves in breast cancer patients with different BCS according to bone metastasis pattern (bone: χ2=366.1, *P*<0.001). **B**. the CSS curves in breast cancer patients with different BCS according to lung metastasis pattern (lung: χ2=182.4, *P*<0.001). **C**. the CSS curves in breast cancer patients with different BCS according to liver metastasis pattern (liver: χ2=135.1, *P*<0.001). **D**. the CSS curves in breast cancer patients with different BCS according to brain metastasis pattern (brain: χ2=32.62, *P*<0.001).

Then, the Kaplan-Meier analyses were used to analyze the prognosis. The results showed that the patients with HR-/HER2- had worst CSS in all metastatic patterns (bone: χ2=366.1, *P*<0.001; lung: χ2=182.4, *P*<0.001; liver: χ2=135.1, *P*<0.001; brain: χ2=32.62, *P*<0.001) (Figure 3A–3D). Interestingly, the patients with the other three BCS did not shown obvious difference on CSS regardless of the metastatic patterns (bone: χ2=2.312, *P*=0.315; lung: χ2=0.761, *P*=0.684; liver: χ2=6.594, *P*=0.037; brain: χ2=1.834, *P*=0.400) (Figure 3A–3D).

### Survival analysis based on different combination metastasis sites

**Figure 4:**
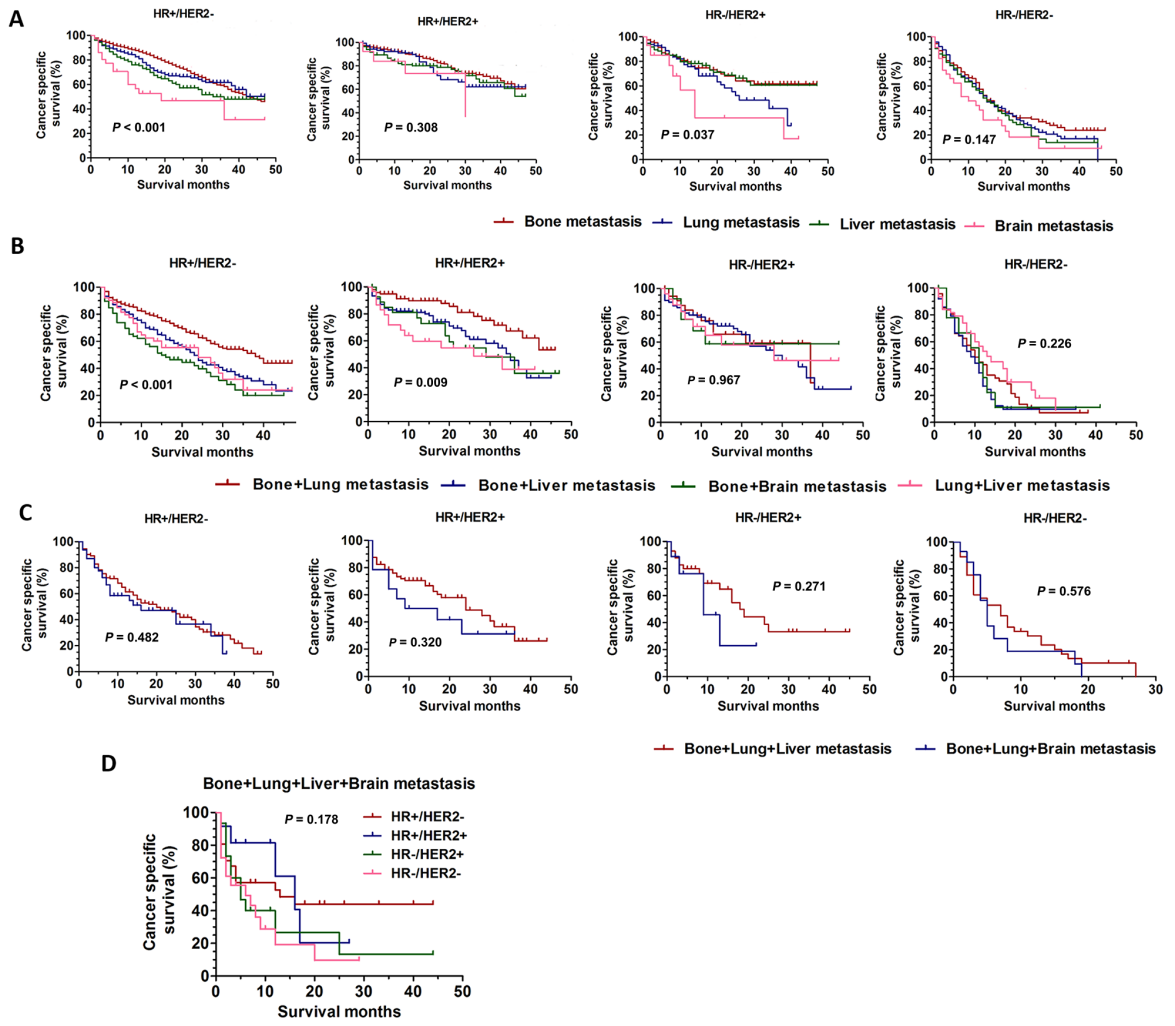
The CSS curves in breast cancer patients with different distant combination metastasis sites **A**. the CSS curves in breast cancer patients with only one distant metastasis site according to different BCS (HR+/HER2-: χ2=29.70, *P*<0.001; HR+/HER2+:χ2=3.610, *P*=0.308; HR-/HER2+:χ2=8.461, *P*=0.037; HR-/HER2-:χ2=5.385, *P*=0.147). **B**. the CSS curves in breast cancer patients with two distant metastasis sites according to different BCS (HR+/HER2-:χ2=33.12, *P*<0.001; HR+/HER2+:χ2=11.49, *P*=0.009; HR-/HER2+:χ2=0.260, *P*=0.967; HR-/HER2-:χ2=4.347, *P*=0.226). **C**. the CSS curves in breast cancer patients with three distant metastasis sites according to different BCS (HR+/HER2-: χ2=0.494, *P*=0.482; HR+/HER2+: χ2=0.990, P=0.320; HR-/HER2+: χ2=1.212, *P*=0.271; HR-/HER2-: χ2=0.313, *P*=0.576). **D**. the CSS curves in breast cancer patients with four distant metastasis sites according to different BCS (χ2=4.918, *P*=0.178).

It has to be noted that many breast cancer patients developed more than one metastatic site. The Kaplan-Meier analyses were used to analyze the prognosis according to different combination metastasis sites. As shown in Figure 4A which represented only one metastasis site, the difference on CSS could be found only in patients with HR+/HER2- (χ2=29.70, *P*<0.001) or HR-/HER2+ (χ2=8.461, *P*=0.0374), and the patients with brain metastasis still had worst CSS. And the difference on CSS was not found in patients with HR+/HER2+ (χ2=3.610, *P*=0.308) or HR-/HER2- (χ2=5.385, *P*=0.147). As shown in Figure 4B which represented two metastasis sites, the patients with bone and lung metastasis had best CSS in HR+/HER2- group (χ2=33.12, *P*<0.001) or HR+/HER2+ group (χ2=11.49, *P*=0.009). However, the difference on CSS was not found in patients with HR-/HER2+ (χ2=0.260, *P*=0.967) or HR-/HER2- (χ2=4.347, *P*=0.226). As shown in Figure 4C, the difference on CSS was not found between the patients with bone, lung and liver metastasis and bone, lung and brain metastasis in all BCS (HR+/HER2-: χ2=0.494, *P*=0.482; HR+/HER2+: χ2=0.990, *P*=0.320; HR-/HER2+: χ2=1.212, *P*=0.271; HR-/HER2-: χ2=0.313, *P*=0.576). Also, the difference on CSS was not found among the four different BCS in patients with four metastasis sites (χ2=4.918, *P*=0.178) (Figure 4D).

## DISCUSSION

The study aimed to better understand the effect of distant metastatic patterns of different BCS on the selection of adjuvant therapy and prediction of survival outcome. Up to date, studies on the association between different BCS and the exact patterns of distant metastasis are limited and inconsistent. It has been reported that the common sites of distant metastatic organs for breast cancer are bone, liver, lung, and brain [6, 7]. Studies have already indicated that bone is the most common distant metastatic organ in breast cancer patients [3, 15, 16]. Consistent with these studies, our results also showed that bone also was the most common metastatic site in all BCS. Interestingly, the predictive value for bone metastasis according to different BCS is still controversial. A recent study have shown that the luminal A (ER+/PR+, HER2-, Ki67<14%) and B subtypes (ER+/PR+, HER2-, Ki67≥14%; ER+/PR+, HER2+, any Ki67) were both significantly associated with bone relapse compared with the HER2 subtype (ER-, PR-, HER2+) and TNBC (ER-, PR-, HER2-) [3]. However, a study from Korean showed that cumulative frequency of bone was not significantly different in patients with different BCS [17]. In our study, we showed that the patients with HR+/HER2− had a significantly higher probability than the patients with other BCS to develop bone metastasis. In fact, most studies have supported the notion that the patients with HR+ (hormone receptor) are more prone to develop bone metastasis [18, 19]. For lung metastasis, a study from China showed that the probability of lung metastasis in patients with TNBC was significantly higher than those in the other three subtypes [16]. Consistent with the studies, our results also showed that the patients with HR-/HER2− had a significantly higher probability than those with HR+/HER2− and HR+/HER2+ to develop lung metastasis. For liver metastasis, the predictive value is also controversial. Some studies have demonstrated that liver metastasis was not associated with BCS [20]. However, some studies demonstrated that liver relapse was more frequently observed in the HER2+ subtype compared with luminal A and TNBC subtypes [3, 16]. The reason for the difference may be attributable to the different sample size. For instance, the study from Korean only included 18 patients with liver metastases [20]. Importantly, a study by Kennecke et al demonstrated that compared with luminal A tumors, luminal/HER2 and HER2-enriched tumors were associated with a significantly higher rate of liver metastases, and the patients with HR−/HER2+ had the highest probability of liver metastasis (23.3%) [21]. Consistent with these studies, our results also showed that the patients with HR-/HER2+ had a significantly higher probability than the patients with the other three BCS to develop liver metastasis. In addition, our results showed that the patients with HR-/HER2− had a significantly higher probability of brain metastasis than the patients with the other three BCS, which was also confirmed by other results [3, 16, 22, 23]. Interestingly, studies found that the patients with TNBC had a relatively higher expression of EGFR [24]. However, higher expression of EGFR increased the risk of brain metastases in breast cancer patients [23]. This may partly explain the reason why the patients with HR-/HER2− had a significantly higher probability of brain metastasis.

Some studies have shown that the differences of the survival in female breast cancer may be linked with different metastatic patterns [7, 25]. However, these studies are controversial and few studies have focused on the survival differences in patients with different metastatic pattern based on different BCS. Some studies have shown that regardless of breast cancer subtypes, 5-year survival rate was significantly higher in patients with mere bone metastasis as compared with other types of local metastasis [25]. However, another study showed the best prognosis was observed among patients with lung as first anatomic site of distant metastasis (58.5 months), followed by those with first metastatic involvement of bone (44.4 months), liver (36.7 months) and brain (7.35 months) [7]. Such differences can be interpreted by the fact that the prognosis was not analyzed based on BCS. In our study, our results demonstrated that the patients with brain metastasis had worst CSS in all BCS groups. However, the patients with bone metastasis have best CSS only if the patients with HR+/HER2- or HR+/HER2+. A study by Eichler et al has demonstrated that the median overall survival from the time of brain metastasis was only 8.3 months; the patients with HR-/HER2− even had a median survival of 4.0 months [26]. As we expected, our results also showed that the patients with HR-/HER2- had worst CSS in all metastatic patterns. This can be mainly explained by the fact that the patients do not benefit from endocrine therapy and targeted therapy. Interestingly, studies showed HER2-positive patients were found to have prolonged survival after brain metastasis compared with HER2-negative patients [26]. However, in our study, we found that only the patients with HR-/HER2+ have a relatively better prognosis in brain metastasis. In fact, studies have shown that targeted drug herceptin can prolong the survival in breast cancer patients with brain metastases [27].

Many breast cancer patients developed more than one metastatic site, and few studies have reported on the combination of metastasis in breast cancer patients. For patients with only one metastasis site including only bone, lung, liver and brain metastasis, we found the overall difference on CSS could be found only in patients with HR+/HER2- or HR-/HER2+. However, for patients with a certain metastasis site including bone, lung, liver and brain metastasis, we found that the overall difference on CSS could be found in all the patients with different BCS. The small sample size of patients with only one brain metastasis site may be an underlying reason for such difference. We found the pattern of brain in combination with other metastasis sites were more frequent in breast cancer patients. Pitifully, the specific mechanism is still not clear. Recent studies have demonstrated that a panel of 22 genes was found to be significantly differentially expressed between primary breast cancer and breast cancer-induced brain metastasis, and brain metastatic cells expressing high levels of c-Met promote the metastatic process via inflammatory cytokine upregulation and vascular reprogramming [28, 29]. In addition, in our study, we also found the most common metastasis involving two sites was different among four BCS. The most common two-site combination metastasis was bone and lung in all the patients with different BCS. The cross-talk between bone microenvironment and lung cancer cells may be an internal mechanism of combination metastasis [30]. We then analyzed the prognosis according to different combination metastasis sites. The results showed that bone and lung metastasis had best CSS only in patients with HR+/HER2- or HR+/HER2+. This may be due to that bone and lung metastases are more likely to benefit from adjuvant therapy, especially endocrine therapy. We then analyzed the prognosis for the patients with three or four metastasis sites. The results also showed that the difference on CSS was not found among the patients with bone, lung and liver metastasis and with bone, lung and brain metastasis or with four metastasis sites. This results may be showed that BCS cannot be a prognostic indicator if the patients existed three or four metastasis sites. In other words, adjuvant therapy may not bring the benefit of survival if the patients had more than three metastases sites. The results of our study may be helpful in the prediction of prognosis and decision of clinical treatment in breast cancer patients.

This study also has several limitations. Firstly, due to the absence of information on chemotherapy or targeted therapy included in the SEER database, their effects on survival could not be evaluated. Secondly, this study is the non-randomized study and the intrinsic defects exist in any retrospective study despite we have a large sample size. Thirdly, in the present study, metastases to the following sites (bone, lung, liver and brain) were only included. Although, the common distant sites for metastasis in breast cancer are bone, liver, lung, and brain, other metastasis sites may influence the prognosis of breast cancer patients.

In conclusion, our study further clarified the relationship between the distant metastatic patterns and the BCS. Importantly, we are the first to perform a prognostic analysis for patients with different distant metastatic pattern based on different BCS.

## METHODS

### Patient selection

The SEER Cancer Statistics Review (http://seer.cancer.gov/data/citation.html) is published annually by the Data Analysis and Interpretation Branch of the National Cancer Institute, MD, USA. A total of 18 population-based cancer registries in the United States were included in the current SEER database [14]. The SEER*Stat software (SEER*Stat 8.3.2) was used to identify the appropriate patients. Using this software, we screened female breast cancer patients between 2010 and 2013. The included patients should meet the following criteria: the diagnosis was confirmed microscopically, they should be female with the confirmed age, active follow-up and only one primary tumor. In addition, only patients with AJCC stage IV were included in this study. Patients with benign or borderline tumors were excluded. And patients lacking information on age, ER/PR, HER2 status, cause of death, unknown survival months were also excluded.

### Ethics statement

This study was mainly based on the SEER database and was conducted in compliance with the Helsinki Declaration. We obtained permission to access the files of SEER program research data and the reference number is 11304-Nov 2015. The informed consent was not required because personal identifying information was not involved. This study was approved by the ethics committee of the Shandong Cancer Hospital affiliated to Shandong University.

### Statistical analysis

For all the patients, the following variables were analyzed: Age, Race, Grade, ER status, PR status, HER2 status, Breast cancer subtype and metastatic site. In addition, the CSS was regarded as the primary endpoint of this study and extracted from the SEER database. The univariate and multivariate logistic regression analysis were used to analyze the association between the BCS and the specific metastatic pattern. In addition, the Kaplan-Meier analyses were used to generate the survival curves and the Log Rank test was applied to analyze the differences among the curves. Comparative risks of mortality were evaluated using univariate and multivariate Cox regression models. All statistical tests were two-sided, and a *P* < 0.05 was considered statistically significant. The statistical software SPSS 18.0 (SPSS, IL, Chicago) was used for all data analysis.

## SUPPLEMENTARY FIGURE


