# Supplementary material for: The prognosis analysis of different metastasis pattern in patients with different breast cancer subtypes: a SEER based study
Source: Oncotarget. 2016 Dec 27;8(16):26368–79. doi: 10.18632/oncotarget.14300 (PMC5432264; doi:10.18632/oncotarget.14300)
Supplement: Supplementary file 1 [file oncotarget-08-26368-s001.pdf]

The prognosis analysis of different metastasis pattern in patients with different breast cancer subtypes: a SEER based study

SUPPLEMENTARY FIGURE

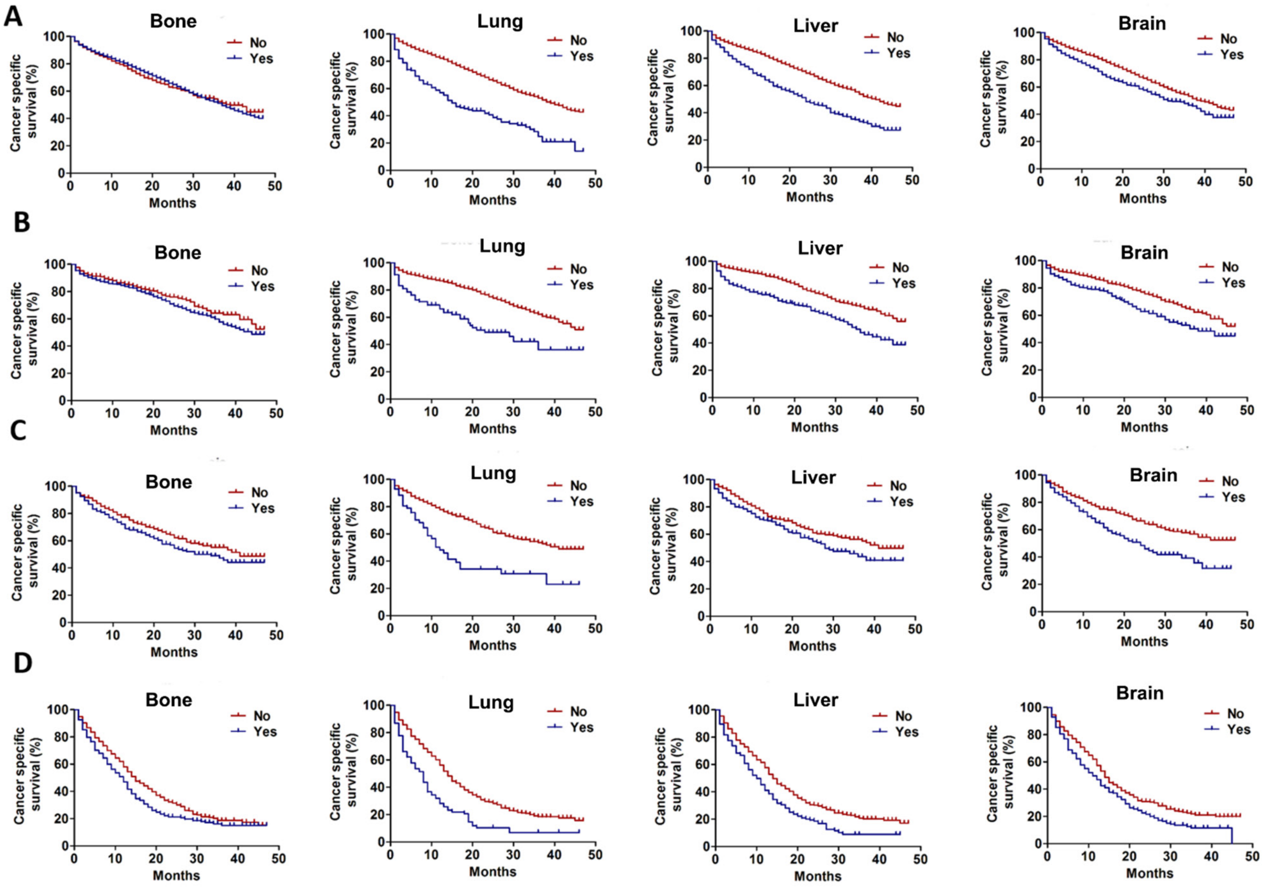

Supplementary Figure 1: The effect of specific metastasis site on CSS in breast cancer patients according different BCS. A. the effect of specific metastasis site on CSS in breast cancer patients with HR+/HER2- (bone: χ2=0.085, *P*=0.770; lung: χ2=111.75, *P*<0.001; liver: χ2=158.71, *P*<0.001; brain: χ2=45.17, *P*<0.001). **B.** the effect of specific metastasis site on CSS in breast cancer patients with HR+/HER2+ (bone: χ2=1.82, *P*=0.177; lung: χ2=20.163, *P*<0.001; liver: χ2=52.81, *P*<0.001; brain: χ2=15.75, *P*<0.001). **C.** the effect of specific metastasis site on CSS in breast cancer patients with HR-/HER2+ (bone: χ2=4.11, *P*=0.043; lung: χ2=28.64, *P*<0.001; liver: χ2=15.58, *P*<0.001; brain: χ2=20.34, *P*<0.001). **D.** the effect of specific metastasis site on CSS in breast cancer patients with HR-/HER2- (bone: χ2=15.10, *P*<0.001; lung: χ2=39.94, *P*<0.001; liver: χ2=29.93, *P*<0.001; brain: χ2=18.54, *P*<0.001).
